# Supplementary material for: A Novel Mechanism for Transcription Termination in the mod(mdg4) Locus of Drosophila melanogaster
Source: Biology (Basel). 2024 Nov 29;13(12):994. doi: 10.3390/biology13120994 (PMC11727487; doi:10.3390/biology13120994)
Supplement: Supplementary file 1 [file biology-13-00994-s001.zip › biology-3327273-supplementary-Table S1.pdf]

#	name of the source vector	procedure	name of resulting vector	comment
1	pAc5.1/V5-His B<br>pGL3-Basic	XbaI (blunt)<br>NcoI (blunt), XbaI (blunt)	**Act-Fluc**	final vector
2.1	pBluescript<br>eGFP cDNA	EcoRV<br>cDNA_d: atggtgagcaagggcgaggagct<br>cDNA_r: cttgtacagctcgtccatgccga	pSK-eGFP	PCR product was cloned into pBluescript. eGFP was in reverse orientation
2.2	pAc5.1/V5-His B<br>pSK-eGFP	KpnI (blunt), SacI(blunt)<br>EcoRI(blunt), HindIII (blunt)	pAc-eGFP	
2.3	pAc5.1/V5-His B<br>pRL	EcoRI (blunt)<br>PstI (blunt), NotI (blunt)	pAc-Rluc	
2.4	pAc-Rluc<br>pAc-eGFP	SalI (blunt), XhoI (blunt)<br>EcoRI (blunt), SalI (blunt)	**Act-Rluc-eGFP**	final control vector
3.1	genomic DNA	ex4: caacagtcccagaactacag<br>Pst_r: acaaacctgcagtagccttagcatcac<br>Pst_d: aggctactgcaggtttgttgttatttat<br>E47_r: aacacagcgcttataaatacaatttttt<br>E47_d: atttataagcgctgtgttatagtaaag<br>Nco_r: ggaaccatggataaaaaaaacacgcaaa<br>Nco_d: ttttttatccatggttccgaattaaagg<br>EcoRI_R: tataagtcgggaattcttcaaggcg<br>EcoRI_d: cgccttgaagaattcccgacttata<br>HpaI_r: ggaagtgttaacatactacaaatatatat<br>HpaI_d: tagtatgttaacacttccggaaaccctaa<br>BamHI_r: taattcggatccaataaaat	intron4(fragment) with restriction sites	The linear product is obtained as a fusion of 6 fragments. Point substitutions were made to create sites for 6 endonucleases.
3.2	pBluescript<br>intron4(fragment) with RS	SalI, BamHI<br>SalI, BamHI	pSK-intron4(fragment)	
3.3	pSK-intron4(fragment)	Eco47III, EcoRI(blunt)	pSK-intron4 ΔEco47III-EcoRI	
3.4	pSK-intron4(fragment)	HindIII(blunt), Eco47III	pSK-intron4 ΔHindIII-Eco47III	
4.1	Act-Rluc-eGFP<br>pSK-intron4(fragment)	EcoRV<br>SphI(blunt), HpaI	**763 bp**	
4.2	Act-Rluc-eGFP<br>pSK-intron4(fragment)	EcoRV<br>SphI(blunt), EcoRI	**603-1198**	

4.3	Act-Rluc-eGFP<br>pSK-intron4(fragment)	EcoRV<br>PstI(blunt), HpaI	**650-1365**	
4.4	Act-Rluc-eGFP<br>pSK-intron4 ΔEco47III-EcoRI	EcoRV<br>PstI(blunt), HpaI	**137 bp + 168 bp**	
4.5	Act-Rluc-eGFP<br>pSK-intron4 ΔHindIII-Eco47III	EcoRV<br>PstI(blunt), HpaI	**Δ723-786**	
4.6	Act-Rluc-eGFP<br>pSK-intron4(fragment)	EcoRV<br>PstI(blunt), Eco47III	**137 bp**	
4.7	Act-Rluc-eGFP<br>genomic DNA	EcoRV<br>115_d: ATTTATGTTTTCAAACTCGT<br>115_r: ATTTTTTTTTTAAGACTCGG	**115 bp**	
4.8	Act-Rluc-eGFP<br>genomic DNA	EcoRV<br>79_d: AAGATGTTGTCTGGATTTGT<br>79_r: AAGACTCGGCAATCAATGCC	**79 bp**	
4.9	Act-Rluc-eGFP	EcoRV<br>synt_sst_d:<br>GGAGTACCACTCAAGCCCATCCGGATGGTAT<br>TTTTGTCCATCAGCGTGGG<br>synt_sst_r:<br>GGAGTCTAATGGCTGGCATCACCATCAAGCC<br>CACGCTGATGGACAAAAAT	**Syntetic**	Oligonucleotides were annealed to each other and extended to form a linear product, followed by cloning into a vector.
4.10	Act-Rluc-eGFP	EcoRV<br>dwil_sst_d: AAGATGTTGTTTTGGTACCTGT<br>GAAAAGGGGTAGCAACGTAAAAG<br>dwil_sst_m: AGGGGTAGCAACGTAAAAGCT<br>TGCTTTTCCCCATTTTGGTTCAA<br>dwil_sst_r AAGACTCGGCAATAAATGCCGTT<br>GTTTGAACCAAAATGGGGAAAAG	**D. willistoni**	Oligonucleotides were annealed to each other and extended to form a linear product, followed by cloning into a vector.
4.11	Act-Rluc-eGFP<br>pAc5.1/V5-His B	EcoRV<br>BamHI(blunt), SalI(blunt)	**PAS SV40**	control vector with PAS from SV40
5.1	pEGFP-N1<br>Act-Rluc-eGFP	BamHI(blunt), KpnI<br>KpnI, NotI (blunt)	**CMV-Rluc-eGFP**	final control vector for mammalian cells

5.2	pEGFP-N1<br>PAS SV40 (Dmel cells)	BamHI(blunt), KpnI<br>KpnI, NotI (blunt)	**SV40**	final control vector for mammalian cells
5.3	pEGFP-N1<br>137 bp (Dmel cells)	BamHI(blunt), KpnI<br>KpnI, NotI (blunt)	**137 bp**	vector for mammalian cells
5.4	pEGFP-N1<br>137 bp + 168 bp (Dmel cells)	BamHI(blunt), KpnI<br>KpnI, NotI (blunt)	**137 bp + 168 bp**	vector for mammalian cells
6.1	white-pAc-Rluc-Fluc-attB *<br>Act-Rluc-eGFP	BamHI, PstI<br>BamHI, PstI	**white-Act-Rluc-eGFP**	final control vector for Dmel transgenesis
6.2	white-Act-Rluc-eGFP<br>PAS SV40 (Dmel cells)	NotI, NheI<br>NotI, NheI	**SV40**	final control vector for Dmel transgenesis
6.3	white-pAc-Rluc-Fluc-attB *<br>137 bp (Dmel cells)	BamHI, PstI<br>BamHI, PstI	**137 bp**	vector for Dmel transgenesis
6.4	white-pAc-Rluc-Fluc-attB *<br>137 bp + 168 bp (Dmel cells)	BamHI, PstI<br>BamHI, PstI	**137 bp + 168 bp**	vector for Dmel transgenesis

* is a laboratory vector containing the white gene, an actin promoter, two luciferases, a polyadenylation signal, and an attB site for integration into the genome
